# Herpes Zoster Ophthalmicus Extending to the Palate

**DOI:** 10.5811/westjem.2014.11.24188

**Published:** 2014-12-05

**Authors:** Todd Schneberk, Edward J. Newton

**Affiliations:** Los Angeles County/University of Southern California Medical Center, Department of Emergency Medicine, Los Angeles, California

A 57-year-old female presented to the emergency department with left sided facial rash with associated pain, blurred vision and oral discomfort. Past medical history included hypertension, and remote scleroderma (untreated). There was no history of neck stiffness, ear pain, environmental exposures, trauma, or immunosuppressive medications. Her facial pain was sharp in quality and extended to her mouth, localizing to her palate. She was mildly hypertensive and other vitals were normal. Physical exam revealed vesicular rash of the left side of her face, along with swelling and periorbital inflammation. There were also multiple vesicular lesions on the left side of her hard palate. Ocular exam showed a small area of fluorescein uptake infranasally concerning for a pseudodendrite, with mild cell and flare of the anterior chamber. There were no vesicles in the ear or tip of her nose.

She was diagnosed with herpes zoster ophthalmicus involving the V1–V2 distribution of the trigeminal nerve, was put on oral acyclovir with opiates for pain control, and referred to ophthalmology. The oral mucosal lesions do not represent another dermatomal involvement, and are extensions of the V2 branch. These lesions are associated with involvement of the palatine nerves, greater and lesser, as well as the nasopalatine nerve, which are extensions of the V2 branch of the trigeminal nerve via the pterygopalatine ganglion.^1^ This distribution of lesions is not uncommon in nonimmunocompromised patients with zoster.^2^ Herpes zoster can also begin on the palate and should be considered in patients presenting with oral lesions and pain.

## Figures and Tables

**Figure:**
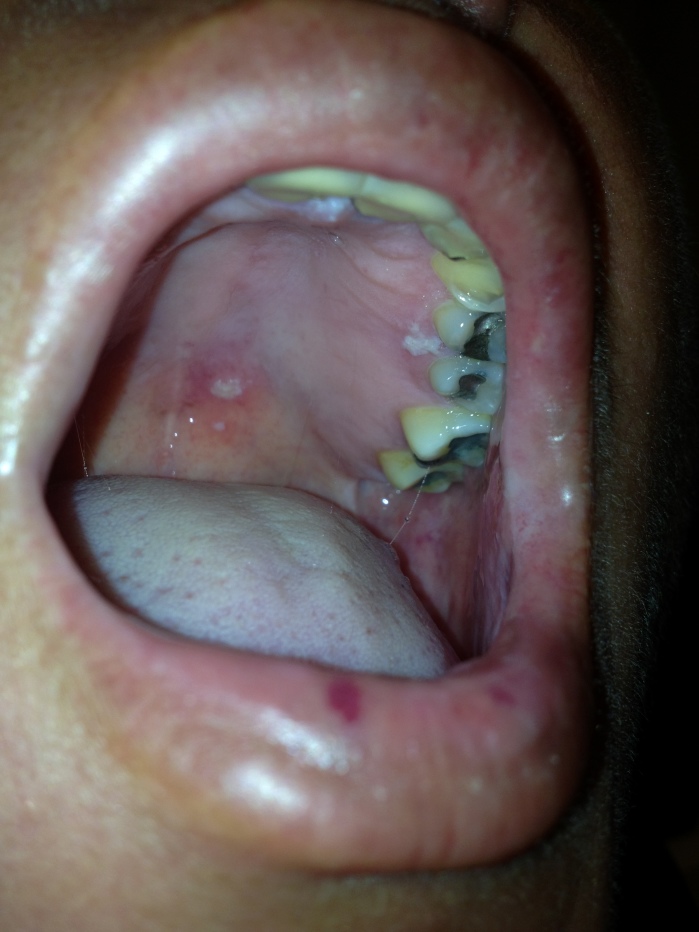
Multiple vesicles on the palate.

## References

[1] Netter Frank H (2006). Atlas of Human Anatomy.

[2] Cohen JI (2013). Clinical practice: Herpes Zoster. N Engl J Med.

